# Microwave Therapy for Cellulite: An Effective Non-Invasive Treatment

**DOI:** 10.3390/jcm11030515

**Published:** 2022-01-20

**Authors:** Luigi Bennardo, Irene Fusco, Cristina Cuciti, Claudia Sicilia, Benedetta Salsi, Giovanni Cannarozzo, Klaus Hoffmann, Steven Paul Nisticò

**Affiliations:** 1 Department of Health Sciences, Magna Graecia University, 88100 Catanzaro, Italy; steven.nistico@gmail.com; 2 Department of Pharmacology, University of Florence, 50121 Florence, Italy; irene.fusco@unifi.it; 3 Unit of Dermatology, San Donato Hospital, 52100 Arezzo, Italy; cristina.cuciti@gmail.com; 4 Department of Adult and Childhood Human Pathology, University of Messina, 98121 Messina, Italy; claudiasiciliakr@gmail.com; 5 Division of Dermatology, Poliambulatorio San Michele, 42121 Reggio Emilia, Italy; slsbdt@gmail.com; 6 Department of Dermatology, Tor Vergata University, 00100 Rome, Italy; drcannarozzo@gmail.com; 7 Department of Dermatology, Ruhr-University, 44787 Bochum, Germany; Klaus.Hoffmann@klinikum-Bochum.de

**Keywords:** cellulite, microwaves, cellulite severity scale, non-invasive therapy

## Abstract

Background: Cellulite represents a common cosmetic problem that affects nearly all women. This study aimed to evaluate microwave therapy’s effectiveness for cellulite treatment. Methods: In this study, 26 women showing severe or moderate cellulite underwent four sessions of microwave therapy on the buttocks and posterior thighs. The following assessments were performed at baseline and the three-month follow-up after the last treatment: the Cellulite Severity Scale (CSS), Nürnberger–Müller classification scale, photographic evaluation, and buttocks/posterior thighs circumference measurements. A Likert scale questionnaire was used to assess patient satisfaction at the 3-month follow-up. Results: The treatment positively affected the cellulite severity as confirmed by the Cellulite Severity Scale (CSS) and Nürnberger–Müller classification scale results. CSS showed a significant amelioration in cellulite severity between the initial assessment and the 3-month follow-up for the buttocks and posterior thighs, with total average scores that ranged from 10.7 ± 3.1 to 4.5 ± 1.8 (*p* < 0.01). The treatment also resulted in a remarkable improvement in comfort/satisfaction and a buttocks and posterior thighs circumference reduction. No serious adverse events were observed. Conclusions: Microwave therapy has proven to be a safe treatment for improving cellulite appearance and reducing body circumferences.

## 1. Introduction

Cellulite, also known as “Oedematous Fibrosclerotic Panniculopathy”, identifies aesthetic changes in the subcutaneous adipose panniculus [1]. The cause of cellulite is not clear; many authors think that it may be considered an endocrine-metabolic microcirculatory disorder that causes structural changes in subcutaneous adipose tissue and interstitial matrix alterations. The most affected areas are the buttocks and thighs [2]. Cellulite is clinically characterized by skin surface alterations, typically raised and depressed lesions (among which depressed lesions are the most frequent and varied in depth and shape), which give the affected areas an “orange peel” aspect [3,4]. The study published by Hexsel and colleagues showed that cellulite depressions are relevantly related to the underlying fibrous septa [5]. Large fibrous septae lead to adipose tissue lobulation determining the typical “orange peel” structure of cellulite.

Today, adiposity and cellulite treatments are two of the most frequent patient requests to the aesthetic doctor and plastic surgeon, especially in this particular historical period. One must also deal with reduced physical activity and lack of access to gyms and fitness centers. A forced sedentary lifestyle often results in a reduction in muscle tone and a lack of muscle stimulation. Nevertheless, if aspiration techniques (liposuction) remain the gold standard for localized fat deposits, skin laxity and cellulite require different techniques and treatments. The classic appearance with wavy or almost knotty skin, typical of the latter, does not disappear with surgical liposuction. This method molds the shapes and volumes, making them more regular but not modifying the skin surface. Numerous therapeutic modalities have been proposed for the treatment of cellulite; among these are cosmetics, therapeutic massage, radiofrequency, ultrasound, laser therapy, carboxytherapy, intense pulsed light, subcision vibration/oscillation platform therapy, and most recently, extracorporeal shockwave therapy (ESWT) or acoustic wave therapy (AWT) [6,7,8,9,10,11].

Unfortunately, the potential for cellulite improvement only in the short-term period despite the numerous treatment sessions limits their popularity. Since the demand for non-invasive and long-lasting treatments to reduce cellulite has grown, new medical devices have been developed as a result. Microwave technology is a non-invasive technique introduced to the market by the ONDA system (DEKA, Florence, Italy), based on microwave propagation into the tissues.

The system produces a connective matrix remodeling of adipose tissue with successive modification of the microenvironment that regulates adipocytes metabolism. Therefore, the homeostatic balance between connective interstitium and adipocytes, responsible for the vitality of the adipose tissue, is altered, inducing metabolic modifications due to thermal stress in the adipocytes themselves, which are stimulated to release several lipids into the environment that surrounds them in quantities much higher than their physiological capacities. As far as cellulite is concerned, however, the energy of the microwaves, absorbed by the fibrous connective septae, causes the solubilization of collagen with resulting debridement of the dense inelastic texture that strangles the adipose lobules [12]. The effect is the loss of the pockmarked appearance of the skin but also a reactivation of the fibroblasts to produce new collagen. Finally, on skin laxity, the heat produced by the microwaves, even rising from the subcutis, causes immediate shrinking (contraction) of the collagen in the dermis, inducing a consequent tightening (tension) [13,14].

It has been demonstrated in previous research that this technology raises cell metabolism and local blood circulation and induces self-regeneration processes increasing elastin fibers and collagen proliferation. From the literature, some studies reported an evident treatment efficacy in cellulite improvement and body contouring after eight sessions with the microwave technology system, allowing professionals to eliminate the “three stones” of the body sculpting procedure (fat, cellulite and wSkin Laxity) in a practical, quick and virtually painless manner without side effects for patients [15,16,17].

Indeed, it was already demonstrated in the study of Zerbinati and colleagues [14] that treatment with ONDA is effective for cellulite through the remodeling of collagen and for skin laxity, with a good tightening of collagen: data have shown that Picrosirius red staining in association with circularly polarized microscopy has had evidence of appreciable changes in the fibrous connective tissue forming the septa. It has been observed that there is a reorganization of the connective tissue; the MMPs (metal proteases, lytic enzymes) digest the old collagen (collagen I), and the fibroblasts, stimulated by this denatured collagen, begin to synthesize new collagen (collagen III), which is more elastic and contributes to the reduction in the orange peel aspect of cellulite. Histological results demonstrate that the treatment is safe (no epidermal–dermal damage was found, showing intact skin), both immediately after treatment and two months after (inflammatory process completely resolved).

In this study, we clinically evaluated and confirmed this non-invasive treatment with the ONDA system for women affected by moderate or severe cellulite.

## 2. Materials and Methods

### 2.1. Patients’ Population

In this study, we reported the clinical results conducted on a group of 26 female patients with cellulite on gluteal and posterior thighs, with a mean age of 38.0 ± 13.1 years and a body mass index (BMI) of 22.6 ± 2.3 kg/m^2^ (see Table 1). Data are represented as means ± standard deviation (SD). All patients completed all scheduled visits. Exclusion criteria were the following: patients who have undergone other aesthetic treatments during the six months prior to the study; patients with past or present heart problems, metabolic disorders, tumors or those who are immunosuppressed; and pregnant or lactating patients. The study was conducted according to the Declaration of Helsinki, and all participants signed and released a written form of consent.

Patients maintained their daily diet and physical activities throughout the entire study period.

### 2.2. Study Protocol

Patients underwent four treatment sessions with ONDA (DEKA, Florence, Italy) system. Sessions were spaced 30 days apart (one session every four weeks).

The ONDA is a microwave platform with two handpieces that produce waves at 2.45 GHz, generating localized, controlled heat absorbed by fat through a biophysical process called “dielectric heating” [15]. The two handpieces are the shallow handpiece (for superficial cellulite and skin tightening) and the deep handpiece (for deep cellulite and targeting fat). The handpieces allow you to “cover” square areas of 15 cm^2^ per side, in a time of about 15 min, with power of up to 200 W according to patient tolerance. The continuous cooling included in the contact handpieces allows the preservation of superficial skin layers from undesirable overheating, ensuring total patient comfort and minimizing side effects and inflammation. Clinical evaluations were performed before the first treatment (at baseline) and three months after the last treatment.

### 2.3. Objective and Patient’s Assessments

#### 2.3.1. Cellulite Severity Scale (CSS) and Nürnberger–Müller Classification Scale

A clinical inspection of the cellulite grade of the patient’s skin was performed. The Cellulite Severity Scale (CSS) and Nürnberger–Müller classification scale evaluated the patient’s cellulite severity.

CSS was proposed by Hexsel et al., 2009 [18] and represented a validated objective and standardized methodology for grading cellulite. It is characterized by five critical morphological aspects of cellulite, which are: depth of depressions; the number of depressions; clinical morphology; degree of laxity, flaccidity or sagging of the skin; and Nürnberger–Müller classification grade. Each variable is graded from 0 to 3, leading to overall grades of mild (1–5), moderate (6–10) and severe (11–15) (see Table 2). The Nürnberger–Müller classification scale [19] has the following grades: grade 0 = no alteration of skin surface; grade I = alterations to skin surface only seen by pinching or contracting the skin; grade II = “orange peel” or “mattress” appearance evident when standing with no skin manipulation; and grade III = findings of grade II plus the presence of raised areas or nodules.

#### 2.3.2. Photographic Evaluation

Standardized digital photographs were performed at baseline and three months after the last treatment, using a digital camera (Reflex Nikon D800, Nikon Corporation, Minato, Tokyo, Japan) to assess cellulite aesthetic clinical improvement.

#### 2.3.3. Buttocks’ and Posterior Thighs’ Circumference Measurements

A circumference measurement was performed on the buttocks at 10 cm below the iliac crest in all patients. Likewise, circumference measurement was performed on each posterior thigh, at 10 cm below the gluteus fold. A flexible but inelastic anthropometric tape was used to take these circumference measurements.

#### 2.3.4. Five-Point Likert Scale Questionnaire

The five-point Likert scale questionnaire (0 = worse; 1 = little satisfaction or not satisfied; 2 = fairly satisfied; 3 = satisfied; and 4 = very satisfied) was used to evaluate patients’ comfort/satisfaction. The five-point Likert scale questionnaire was used to assess patient satisfaction at 3-monthfollow-up (MFU).

#### 2.3.5. Side Effects

Possible side effects were monitored and evaluated, such as pain, itching, soreness, redness, swelling, burns, nodules, post-operative ecchymosis, edema or blisters.

### 2.4. Statistical Analysis

Student’s *t*-test and SPSS 26.0 (IBM Corp., New York, NY, USA) were used to perform statistical analysis. Data are represented as means ± standard deviation (SD).

### 2.5. Post-Treatment Protocol

The treatment can be followed by a lymphatic drainage massage by an independent operator. It is recommended that patients avoid direct exposure to the sun for 1–2 days after the session and avoid washing the treated area with hot water in case of erythema. Finally, the patient should drink about 2 L of water a day to promote the drainage of interstitial fluids, thus avoiding the risk of dehydration during the session.

## 3. Results

### 3.1. Cellulite Severity Scale (CSS) and Nürnberger–Müller Classification Scale

**Figure 1:**
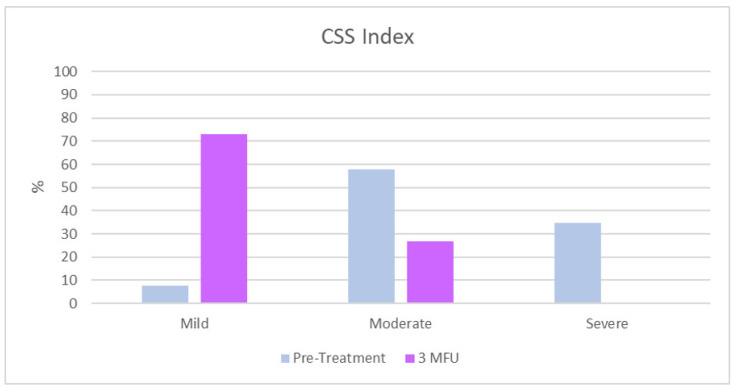
Cellulite Severity Scale (CSS) at baseline and 3MFU after the last session of ONDA.

CSS analysis has shown a significant improvement in the severity of cellulite between the initial assessment and the 3-month follow-up after the last treatment for the buttocks and posterior thighs. The total mean CSS (±SD) at baseline was 10.7 ± 3.1. A total of 57.7% out of the 26 patients (*n* = 15) showed a moderate CSS value, followed by 34.6% of women with a severe CSS value, (*n* = 9) and only 7.7 % (*n* = 2) patients had a mild CSS value. At 3MFU after the last treatment, all the parameters examined according to the CSS were improved (*p* ˂ 0.01). The total mean CSS was 4.5 ± 1.8. 73.1% (*n* = 19) for the women who showed a mild CSS value, while 26.9% (*n* = 7) manifested a moderate CSS index. A total of 0% (*n* = 0) of the women showed a severe CSS value. (Table 3 and Figure 1).

The average number of depressions significantly decreased (2.2 ± 0.7 at baseline vs. at 0.9 ± 0.3 3MFU after last treatment, *p* ˂ 0.01). The average depth of depressions significantly decreased (2.1 ± 0.9 at baseline vs. 0.8 ± 0.5 at 3MFU after the last treatment, *p* ˂ 0.01). The morphological appearance of skin surface alterations significantly improved (2.1 ± 0.8 at baseline vs. 0.8 ± 0.4 at 3MFU after last treatment, *p* ˂ 0.01). Additionally, the grade of laxity, flaccidity or sagging skin (2.1 ± 0.7 at baseline vs. 1.0 ± 0.6 at 3MFU after last treatment, *p* ˂ 0.01) and the Nürnberger–Müller classification (2.2 ± 0.6 at baseline vs. 1.0 ± 0.6 at 3MFU after last treatment, *p* ˂ 0.01) significantly decreased (see Table 4).

**Figure 2:**
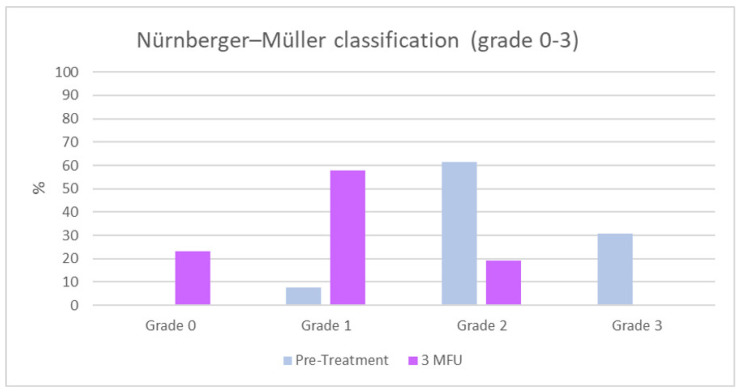
Nürnberger–Müller classification (grade 0–3) at baseline and 3MFU after the last session of ONDA.

According to the Nürnberger and Müller classification, at baseline, 0% (*n* = 0) of the patients showed a grade 0, 7.7% (*n* = 2) of the patients showed grade I, 61.5% (*n* = 16) grade II and 30.8% (*n* = 8) grade III cellulite. An overall improvement of the cellulite grade was observed at the 3MFU after the last treatment, with a significant increase in the percentage of women with grade I/0 and a significant reduction in the percentage of patients who had grade II/III cellulite (23.1% (*n* = 6) patients with grade 0, 57.7% (*n* = 15) patients with grade I, 19.2% (*n* = 5) patients with grade II and 0%(*n* = 0) patients with a grade III cellulite, 3MFU) as shown in Table 5 and Figure 2.

### 3.2. Photographic Evaluation

**Figure 3:**
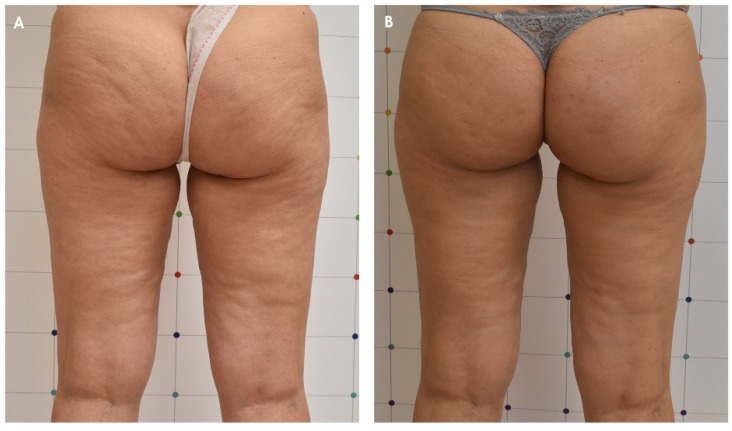
Improvement of the cellulite of a female patient before (**A**) and 3 months after the last treatment with ONDA (**B**).

**Figure 4:**
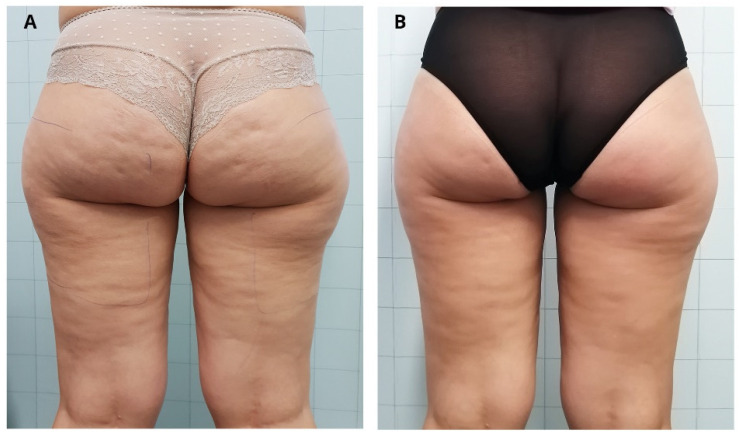
Improvement of the cellulite of a female patient before (**A**) and 3 months after the last treatment with ONDA (**B**).

Nürnberger and Müller’s classification and the CSS results as well as the photographic evaluation confirmed the improvement of cellulite appearance, as shown in Figure 3 and Figure 4.

### 3.3. Patient Satisfaction Index with Five-Point Likert Scale Questionnaire

Patients reported a significant improvement of cellulite. Based on a score of 0–4, patients agreed (3.7 ± 0.4) that they were satisfied with the treatment results.

### 3.4. Buttocks and Posterior Thighs Circumference Measurements

All patients observed a reduction in the total mean circumference of the gluteal area (from 99.5 ± 5.1 cm at baseline to 95.2 ± 5.6 cm at 3MFU after the last treatment). Furthermore, a total mean reduction in the posterior thighs’ circumferences was observed at the measurement point 10 cm below the gluteus fold (from 49.5 ± 3.1 cm at baseline to 47.4 ± 3.2 cm at 3MFU after the last treatment).

### 3.5. Side Effects

None of the patients experienced pain (at most, the patient reported a slight tingling/sensation of contraction) or other side effects during the treatment or the follow-up period. However, it was possible to observe a temporary mild redness on the skin due to the passage of microwaves that cause subcutaneous heating, which resolved within a few days.

## 4. Discussion

Over the latest 30 years, there has been a significant increase in the demand for non-invasive methods for use in the medical and aesthetics sectors to treat skin imperfections in general and in particular, to treat localized adiposity cellulite treatment and skin laxity. RF techniques are known to be one of the most common therapies for cellulite. Indeed, several companies suggest RF systems as valid and safe alternative technologies capable of achieving the same clinical results in improving cellulite [20]. Despite this evidence for RF devices in treating cellulite, no procedure has been successful in the long-term period [21]. Microwaves are part of the RF spectrum (frequency range between 1 and 300 GHz) [22], and this technology has become very popular in modern society and is not even a newcomer to medical applications. It has been used extensively in many branches of medicine until now, including Surgery, Oncology and Dermatology. The application of 2.45 GHz non-invasive, high-energy microwaves to the body directs a targeted action towards the subcutaneous adipose tissues to promote the heating of the adipocyte cells without affecting the dermal–epidermal layers. This process leads to a complete metabolic macrophage adipolysis compatible with subcutaneous adipose tissues reduction and a consequent circumference diminution. Additionally, cellulite is improved by the solubilization of collagen septae caused by subcutaneous adipose tissue heating, which causes dermal collagen fibers’ contraction and improves external skin architecture [23,24].

The solubilization of the deeper collagen fibers, the activation of fibroblasts and the collagen fibers’ remodeling could be caused by controlled hyperthermia [15]. On these bases, by evaluating ONDA’s safety and long-term efficacy in the treatment of cellulite, we have found and reported a significant amelioration of cellulite with a significant decrease in the number and depth of depressions in all subjects treated. The mean total CSS score improved from moderate-severe at baseline to mild at the 3MFU after the last protocol treatment with ONDA. Furthermore, patients’ cellulite appearance amelioration was confirmed by the improvement of grade scores obtained with the Nürnberger and Müller classification scale analysis. Additionally, the treatment’s effects on body circumference showed promising results, reducing buttocks and posterior thighs circumferences.

In addition, the evaluation of patient satisfaction levels through the five-point Likert scale questionnaire and the photographic assessment corroborates these results.

No side effects were observed, neither after the single treatment sessions nor after the entire follow-up period, demonstrating that the treatment is safe and well supported by all patients. The protocol was found to be safe and non-invasive for the patient, who can experience at most a slight tingling/sensation of contraction or a possible temporary redness of the skin, due to the passage of microwaves that cause subcutaneous heating.

Limitations of this study included the fact that circumference measures are difficult to achieve accurately, as well as a relatively low number of study participants.

The great advantage of ONDA is that this high degree of innovation has provided a revolutionary non-invasive, no-consumables platform with fast results in a highly controlled and safe way. The patented handpiece technology provides users with maximum control on the depth of penetration so that the vital organs are not affected and make the entire procedure extremely safe for both the patient and operator. With ONDA, unlike other therapies for body remodeling and cellulite, which require longer times and a higher number of sessions, and in comparison to the research of Di Pietro and colleagues [15] on the same device, in this study of 3–4 treatments, the results of cellulite improvement are visible, both regarding the reduction in skin laxity, =the typical “orange peel” aspect, and the decrease in body circumferences. ONDA represents, for this reason, a completely different universe in the body-shaping device market.

## 5. Conclusions

Our results confirmed the safety and effectiveness of ONDA therapy in treating cellulite for the buttocks and posterior thighs in a long-term period, without having found any adverse effects.

## Figures and Tables

**Table 1:** Demographic data. BMI: body mass index. CSS: Cellulite Severity Scale.

N° of patients	26
Mean age (years; mean ± SD)	38.0 ± 13.1
BMI (Kg/m^2^; mean ± SD)	22.6 ± 2.3
Smoking habits *n* (%)	0 (0)
Contraceptive methods *n* (%)	13 (50)
Pregnancy history *n* (%)	16 (60)
CSS *n* (%) Mild Moderate Severe	2 (7.7) 15 (57.7) 9 (34.6)

**Table 2:** Cellulite Severity Scale (CSS) of mild, moderate or severe degree.

Cellulite Severity Scale	Classification
1–5	Mild
6–10	Moderate
11–15	Severe

**Table 3:** Mean patient differences in CSS percentages before and 3MFU (3-monthfollow-up) after the last treatment.

CSS *n* (%)	Pre-Treatment (*n* = 26)	Post-Treatment (*n* = 26)
Mild	2 (7.7)	19 (73.1)
Moderate	15 (57.7)	7 (26.9)
Severe	9 (34.6)	0 (0)

**Table 4:** Cellulite Severity Scale (CSS) mean scores of each key morphologic aspects of cellulite before and 3MFU after the last treatment in the two body areas treated.

CSS Key Morphologic Aspects of Cellulite	Pre-Treatment Posterior Thigh/Buttocks (*n* = 26)	Post-Treatment (3MFU) Posterior Thigh/ Buttocks (*n* = 26)	*p* Value
Average number of depressions	2.2 ± 0.7	0.9 ± 0.3	*p* ˂ 0.01
Average depth of depressions	2.1 ± 0.9	0.8 ± 0.5	*p* ˂ 0.01
Average morphological appearance of skin surface alterations	2.1 ± 0.8	0.8 ± 0.4	*p* ˂ 0.01
Average grade of laxity, flaccidity or sagging skin	2.1 ± 0.7	1.0 ± 0.6	*p* ˂ 0.01
Nürnberger–Müller classification	2.2 ± 0.6	1.0 ± 0.6	*p* ˂ 0.01
CSS mean total score	10.7 ± 3.1	4.5 ± 1.8	*p* ˂ 0.01

**Table 5:** Cellulite Severity Scale (CSS) of mild, moderate or severe degrees.

Cellulite Grades	Pre-Treatment (*n* = 26)	Post-Treatment (*n* = 26)
Grade 0, *n* (%)	0 (0)	6 (23.1)
Grade 1, *n* (%)	2 (7.7)	15 (57.7)
Grade 2, *n* (%)	16 (61.5)	5 (19.2)
Grade 3, *n* (%)	8 (30.8)	0 (0)

## Data Availability

Data that support the study findings are available on request from the corresponding author.

## References

[1] Rossi A., Vergnanini A. (2000). Cellulite: A review. J. Eur. Acad. Dermatol. Venereol..

[2] Draelos Z., Marenus K. (1997). Cellulite. Etiology and purported treatment. Dermatol. Surg..

[3] Segers A., Abulafia J., Kriner J., Cortondo O. (1984). Celulitis. Estudo histopatológico e histoquímico de 100 casos. Med. Cut. ILA.

[4] Scherwitz C., Braun-Flaco O. (1978). So-called cellulite. J. Dermatol. Surg. Oncol..

[5] Hexsel D.M., Abreu M., Rodrigues T.C., Soirefmann M., do Prado D., Gamboa M.M.L. (2009). Side-By-Side Comparison of Areas with and without Cellulite Depressions Using Magnetic Resonance Imaging. Dermatol. Surg..

[6] Sadick N. (2019). Treatment for cellulite. Int. J. Womens Dermatol..

[7] Hexsel D., Camozzato F.O., Silva A.F., Siega C. (2017). Acoustic wave therapy for cellulite, body shaping and fat reduction. Clin. Trial J. Cosmet. Laser Ther..

[8] Uebel C.O., Piccinini P.S., Martinelli A., Aguiar D.F., Ramos R.F. (2018). Cellulite: A surgical treatment approach. Aesthetic Surg. J..

[9] Rawlings A.V. (2006). Cellulite and its treatment. Int. J. Cosmet. Sci..

[10] Writers A.M. (2015). Cellulite: No clear evidence that any type of treatment is effective. Drugs Ther. Perspect..

[11] Modena D.O., da Silva N.C., Delinocente T.C.P., de Araújo T.B., de Carvalho T.M., Grecco C., Moreira R.G., Campos G., de Souza J.R., Guidi R.M. (2019). Effectiveness of the Electromagnetic Shock Wave Therapy in the Treatment of Cellulite. Dermatol. Res. Pract..

[12] Foster K.R., Ziskin M.C., Balzano Q. (2016). Thermal Response of Human Skin to Microwave Energy: A Critical Review. Health Phys..

[13] Toker S., Boone-Kukoyi Z., Thompson N., Ajifa H., Clement T., Ozturk B., Aslan K. (2016). Microwave Heating of Synthetic Skin Samples for Potential Treatment of Gout Using the Metal-Assisted and Microwave-Accelerated Decrystallization Technique. ACS Omega.

[14] Zerbinati N., D’Este E., Farina A., Cornaglia A.I., Jafferany M., Golubovic M., Binic I., Sigova J., Van Thuong N., Tirant M. (2020). Remodeling of collagen constituting interlobular septa of subcutaneous adipose tissue following microwaves application. Dermatol. Ther..

[15] Bonan P., Marini L., Lotti T. (2018). Microwaves in body sculpting: A prospective study. Dermatol. Ther..

[16] Di Pietro A., Ferri S., Bonan P., Verdelli A., Stevan S., Tartaglia C., Perosino E. (2019). Effectiveness of microwaves in the treatment of cellulite: A preliminary study. J. Plastic Pathol. Dermatol..

[17] Bonan P., Verdelli A. (2021). Combined microwaves and fractional microablative CO2 laser treatment for postpartum abdominal laxity. J. Cosmet. Dermatol..

[18] Hexsel D., Dal’Forno T., Hexsel C. (2009). A validated photonumeric cellulite severity scale. J. European Acad. Dermatol. Venereol..

[19] Nürnberger F., Müller G. (1978). So-Called Cellulite: An Invented Disease. J. Dermatol. Surg. Oncol..

[20] Belenky I., Margulis A., Elman M., Bar-Yosef U., Paun S.D. (2012). Exploring Channeling Optimized Radiofrequency Energy: A Review of Radiofrequency History and Applications in Esthetic Fields. Adv. Ther..

[21] Da Silva R.M.V., Arend Barichello P., Lima Medeiros M. (2013). Effect of Capacitive Radiofrequency on the Fibrosis of Patients with Cellulite. Dermatol. Res. Pract..

[22] Beasley K.L., Weiss R.A. (2014). Radiofrequency in cosmetic dermatology. Dermatol. Clin..

[23] Pahlavani N., Nattagh-Eshtivani E., Amanollahi A., Ranjbar G., Aghdaei H.A., Navashenaq J.G., Shabaninezhad Z., Sharahi N.R., Maleki M., Malekahmadi M. (2021). Effects of microwave technology on the subcutaneous abdominal fat and anthropometric indices of overweight adults: A clinical trial. J. Cosmet. Dermatol..

[24] Kennedy J., Verne S., Griffith R., Falto-Aizpurua L., Nouri K. (2015). Non-invasive subcutaneous fat reduction: A review. J. Eur. Acad. Dermatol. Venereol..

